# Toll-like receptor 4 deletion partially protects mice from high fat diet-induced arterial stiffness despite perturbation to the gut microbiota

**DOI:** 10.3389/frmbi.2023.1095997

**Published:** 2023-05-23

**Authors:** Kayl E. Ecton, Elliot L. Graham, Briana D. Risk, Gabriele D. Brown, Grace C. Stark, Yuren Wei, S. Raj J. Trikha, Tiffany L. Weir, Christopher L. Gentile

**Affiliations:** 1 Cell and Molecular Biology Graduate Program, Colorado State University, Fort Collins, CO, United States; 2 Department of Microbiology, Immunology, and Pathology, Colorado State University, Fort Collins, CO, United States; 3 Department of Food Science and Human Nutrition, Colorado State University, Fort Collins, CO, United States; 4 University of Colorado School of Medicine at Colorado State Univeristy, Fort Collins, CO, United States

**Keywords:** gut microbiota, lipopolysaccharides (LPS), arterial stiffness, pulse wave velocity, toll like receptor 4

## Abstract

The present study aimed to determine the effects of toll-like receptor 4 (TLR4) deletion on high fat diet-induced aortic stiffness and gut microbiota alterations. We hypothesized that a high fat diet would result in perturbation of the gut microbiota in both control and TLR4 knockout mice (TLR4^−/−^), but that the absence of TLR4 signaling would protect mice from downstream vascular consequences of the high fat diet. Male control mice (CON, n=12) and TLR4^−/−^ mice (KO, n=12) were fed either a standard low-fat diet (SD) or a high fat diet (HFD) (60% kcals from fat) for 6 months, after which time measurements of aortic stiffness (via pulse wave velocity [aPWV]) and gut microbiota composition (16S rRNA sequencing) were determined. Compared to the SD, HFD reduced microbial variability, promoted perturbation of the gut microbiota, and increased intestinal permeability in both CON and KO mice, with no effect of genotype. This increased intestinal permeability in HFD mice was accompanied by increases in plasma lipopolysaccharide binding protein (LBP) levels, an indicator of circulating endotoxin (p<0.05 for all comparisons between HFD and SD groups). aPWV was increased in CON+HFD mice (CON+HFD vs CON+SD: 525.4 ± 16.5 cm/sec vs. 455.2 ± 16.5 cm/sec; p<0.05), whereas KO+HFD mice displayed partial protection from HFD-induced arterial stiffening (KO+HFD vs. CON+SD: 488.2 ± 16.6 cm/sec vs. 455.2 ± 16.5 cm/sec; p=0.8) (KO+HFD vs. CON+HFD: 488.2 ± 16.6 cm/sec vs. 525.4 ± 16.5 cm/sec; p=0.1). In summary, TLR4 KO mice are not protected from deleterious alterations in gut microbial composition or intestinal permeability following a HFD, but are partially protected from the downstream arterial stiffening, suggesting that TLR4 signaling is not required for HFD-mediated intestinal disturbances, but is an important determinant of downstream vascular consequences.

## Introduction

Obese individuals are more than twice as likely as non-obese individuals to develop cardiovascular disease (CVD) during their lifetime (Eckel and Krauss, 1998). One key process that links obesity to CVD is the development of vascular dysfunction, which can clinically manifest as stiffness of the large elastic arteries. As such, arterial stiffness is an independent predictor of future CVD events and mortality (Vlachopoulos et al., 2010). Unhealthy dietary patterns, including consumption of a high fat diet, are implicated in multiple cardiometabolic disruptions, such as glucose intolerance, systemic inflammation, and arterial stiffness (David et al., 2014; Brown and Hazen, 2015; Battson et al., 2018). High fat diets have also been shown to profoundly impact the gut microbiome, a diverse ecosystem consisting of trillions of bacteria within the gastrointestinal tract that collectively impact nearly every facet of mammalian metabolism (Ojeda et al., 2016). Numerous studies, including those from our own laboratory, have suggested that diet-induced changes to the gut microbiome may play a role in downstream cardiometabolic impairments (Qiao et al., 2013; Battson et al., 2018; Lee et al., 2020).

Although multiple signaling pathways are involved in diet-microbiota-host crosstalk, Cani and colleagues (Cani et al., 2007a; Cani et al., 2008) proposed that sub-clinical levels of circulating bacterial lipopolysaccharides (LPS) provide a key mechanistic link between the microbiota, obesity, and downstream disease development. Chronic consumption of a high fat diet can lead to this physiologic state, also known as “metabolic endotoxemia”, by causing gut microbiota perturbations, characterized by disrupted microbial communities, increased intestinal inflammation, loss of epithelial barrier integrity and translocation of luminal contents into the circulation. LPS can also translocate to the circulation through incorporation into mixed micelles after ingestion of a high fat meal (Laugerette et al., 2011; Clemente-Postigo et al., 2012; Ryu et al., 2017; Fuke et al., 2019). Once in circulation, LPS binds to Toll-like receptor 4 (TLR4) on various cell types to induce a signaling cascade that culminates in inflammation and generation of reactive oxygen species that can drive development of cardiometabolic dysfunction, including arterial stiffness (Ciesielska et al., 2021).

Our laboratory has observed that the gut microbiota is a critical regulator in the development of obesity-associated arterial stiffness. Specifically, we have demonstrated that antibiotic treatment ameliorates arterial stiffness in western diet-fed mice (Battson et al., 2018) and that transplantation of an obesity-associated microbiota from obese mice (Battson et al., 2019) or obese humans (Trikha et al., 2021) is sufficient to increase arterial stiffness in lean mice. In these experiments, increased arterial stiffness was often associated with decreased intestinal barrier function and/or evidence of increased LPS signaling (Battson et al., 2018; Battson et al., 2019). These data led us to further examine the role of TLR4-mediated inflammation in vascular dysfunction by examining the cardiometabolic effects of a high fat diet in TLR4 knockout mice (TLR4^−/−^). We hypothesized that TLR4^−/−^ mice fed a high fat diet would manifest microbial disruption and impaired gut barrier function like wild-type animals, but given the proinflammatory effects of TLR4 signaling, TLR4^−/−^ mice would be protected from downstream cardiometabolic derangements.

## Methods

### Experimental design

Eight-week-old male TLR4^−/−^ (KO) and TLR4^+/+^ littermates (CON) were obtained from The Jackson Laboratory (Bar Harbor, ME) and acclimated for two weeks with *ad libitum* access to a purified standard diet (SD; Harlan Laboratories, TD 08485) consisting of 13% fat, 67.9% carbohydrate, and 19.1% protein (% kcal). After two weeks, animals were assigned to either a standard low-fat diet (SD) or a high fat diet (HFD; Harlan Laboratories, TD 06414) consisting of 60.3% fat, 21.4% carbohydrate, and 18.3% protein (% kcal), resulting in the following 4 experimental groups (n=12 per group): 1) TLR4^+/+^ on SD diet (CON+SD); 2) TLR4^+/+^ on HFD diet, (CON+HFD); 3) TLR4^−/−^ on SD diet (KO+SD); and 4) TLR4^−/−^ on HFD diet (KO+HFD). Mice remained on assigned diets through 6 months and were cohoused two per cage in a temperature and humidity-controlled environment on a 12:12-h light-dark cycle. Food intake and body weight were measured weekly at the time of cage and water changes. All animal procedures were reviewed and approved by the Colorado State University Institutional Animal Care and Use Committee protocol #1369.

### Arterial stiffness

Aortic pulse wave velocity (aPWV) was used to determine *in vivo* arterial stiffness using a Doppler Flow Velocity System (Indus Instruments, Webster, TX) via methods described previously by our laboratory (Battson et al., 2018; Lee et al., 2018). aPWV (in cm/s) was calculated as aPWV = (distance between the 2 probes)/(Δtime_abdominal_ − Δtime_carotid_).

### Glucose and insulin tolerance tests

Following a 6h fast, mice received an intraperitoneal injection of 2g/kg dextrose, and blood glucose was assessed at 0, 15, 30, 45, 60, 90, and 120 minutes post-injection using tail-vein blood and AlphaTRAK 2 glucometers (Abbott Laboratories, Chicago, IL). Fasting blood was spun for 5 minutes at 2,000rpm and resultant serum was used for fasting insulin levels via ELISA. For insulin tolerance tests, mice received an intraperitoneal injection of 1.5g/kg insulin after a 6h fast, and blood glucose was assessed at 0, 15, 60, and 120 minutes post-injection as described above.

### *In vivo* intestinal barrier integrity

Animals were water-fasted for 12 hours prior to oral gavage administration of fluorescein isothiocyanate (FITC)-dextran (4,000 mol wt, no. 46944; Sigma-Aldrich; 125 mg/ml for SD fed mice and 150 mg/ml for HFD fed mice). Food was removed immediately after oral gavage and tail vein blood samples were collected after 4 hours for quantification of serum FITC-dextran concentrations. Serum samples were diluted 1:2 in 1X PBS, and fluorescence was measured on a BioTek Synergy 2 Multi-Detection Microplate Reader (BioTek Instruments, Winooski, VT) at 485/20 (excitation) and 528/20 (emission). Serum concentrations were quantified using a standard curve of known FITC-dextran concentrations prepared in a control serum sample from an untreated mouse.

### Animal termination and tissue collection

Mice were anesthetized with isoflurane and euthanized by exsanguination via cardiac puncture. Blood was collected in a 0.5 M EDTA-coated syringe (no. 15575020; Invitrogen). Collected blood was added to 2% volume EDTA and plasma was obtained by centrifugation at 2,000 rcf for 10 min at 4°C, and immediately flash frozen in liquid nitrogen. The gastrointestinal tract was excised, and colon length and cecum weight were recorded. Cecal content and colon content were removed from the tissue and flash frozen. Adipose tissue depots (perivascular, subcutaneous, epididymal, and mesenteric depots) were isolated and weighed. Sections of distal colon and proximal small intestine were obtained and flash frozen for further analysis.

### Biomarker analysis

Circulating biomarkers associated with intestinal permeability were evaluated in plasma collected by cardiac puncture. Specifically, circulating levels of LPS binding protein (LBP; ALX-850-304/1, Enzo Life Sciences) and zonulin-family peptides (NC1314884, Fisher Scientific) were measured by ELISA. LBP is a soluble acute phase protein that binds and presents LPS to TLR4 and its co-receptor CD14. Zonulin-family peptides are potent regulators of intestinal tight junctions and circulating levels have been associated with “leaky gut”, inflammation, and gut microbiota perturbation (Tajik et al., 2020). Additionally, secretory IgA (sIgA; 50154425, Fisher Scientific) and intestinal alkaline phosphatase (IPA; NC9945170, Fisher Scientific) were measured by ELISA in collected ileal tissues. sIGA and IAP were normalized to total protein, measured by BCA. Intestinal sIgA is secreted in response to luminal pathogens or enterotoxins and IAP dephosphorylates bacterial LPS, reducing its immunogenicity and reduced IAP is associated with loss of gut homeostasis.

### DHE staining and analysis

Thoracic aorta (TA) was excised and placed in an ionized solution for removal of perivascular adipose tissue (PVAT). After PVAT removal, a 1–1.5mm section distal to the aortic arch was cut and embedded vertically in optimal cutting temperature (OCT) gel that was flash frozen. Sections were cut at 10μm using a cryostat set to −20°C. 4–5 sections were adhered to each slide and 3–5 slides per mouse were stored at −80°C until staining. 50ml fresh 5 μM DHE (D11347, Thermo Fisher Scientific) staining solution prepared and poured in slide box. 1mg DHE dissolved into 317 μL dimethyl sulfoxide (DMSO) for 10mM stock solution. 25μL DHE stock diluted into 50 mL Milli-Q pure water for 5μM staining solution. Slides were rinsed then placed in DHE staining solution and incubated for 15 min at room temperature, with limited light exposure. Slides were washed and fluorescent images were acquired. Two to three technical replicates were collected on 2–3 biological replicates for each mouse and replicates were averaged for one mean grey intensity value/area (μm^2).

### Microbiota characterization

Fecal DNA was extracted from samples excised from the colon at termination using the FastDNA Stool kit (Catalog 6540400, MP Biomedicals). PCR amplification of the V4 16S rRNA region was completed following the Earth Microbiome Project protocol using the 515F-806R fusion primers with 12bp Golay barcodes and sequencing adaptors (https://earthmicrobiome.org/protocols-and-standards/16s/). Pooled libraries were sent to the Next Generation Sequencing Facility at Colorado State University and sequenced using 2×250 chemistry on a MiSeq (Illumina Inc, San Diego, CA).

Forward and reverse reads were imported into QIIME2 version 2021.2. Reads were demultiplexed, concatenated, and trimmed to 200 base pairs. Reads were binned into ASVs using the DADA2 pipeline and taxonomic assignments were made using GreenGenes version 13.8. Count tables and taxonomy were imported into MicrobiomeAnalyst (www.microbiomeanalyst.ca) and further curated by removing reads that had fewer than 4 counts and that were present in less than 10% of the samples, which resulted in the removal of 16 low abundance features.

### Statistical analyses

Data are presented as either mean ± standard deviation (SD), mean ± standard error of the mean (SEM), or as median (interquartile range (IQR)). Data analysis and visualization was conducted in GraphPad Prism (version 9). Before statistical analysis, identification and removal of outliers was performed using the ROUT test or Grubbs’ test available in GraphPad Prism. To test for differences in outcomes over time, we fit two-way repeated measures ANOVA, followed by Tukey’s multiple comparisons tests, if indicated. If observations were missing at random time points, we fit linear mixed effect models with individual mice as a random effect and week and group as fixed effects, followed by Tukey’s multiple comparisons tests if indicated. To test for differences in outcomes not measured over time, we fit one-way ANOVAs follwed by Tukey’s multiple comparisons tests if indicated. Either Welch ANOVA models followed by Dunnett’s T3 multiple comparisons tests or Kruskal Wallis tests followed by Dunns multiple comparisons tests were fit if data violated the assumption of homoscedasticitiy or normality, respectively. Microbiome data were statistically analyzed using Kruskal-Wallis test with FDR-corrected (Benjamini, Krieger, Yekutieli method) *post-hoc* analysis when indicated. Beta-diversity ordination ellipses show the 95% confidence interval for each group and statistical evaluation was conducted using PERMANOVA and PERMDISP to examine differences in centroids and heterogeneity between groups, respectively (Anderson, 2001). Heat trees provided between group comparisons and analyzed using non-parametric Wilcoxon test. LEfSe was used to identify taxa that serve as biomarkers for the different experimental conditions (Segata et al., 2011). A p-value ≤ 0.05 and LDA>2.0 was considered statistically significant.

## Results

### TLR4 KO mice are partially protected from diet-induced weight gain

**FIGURE 1:**
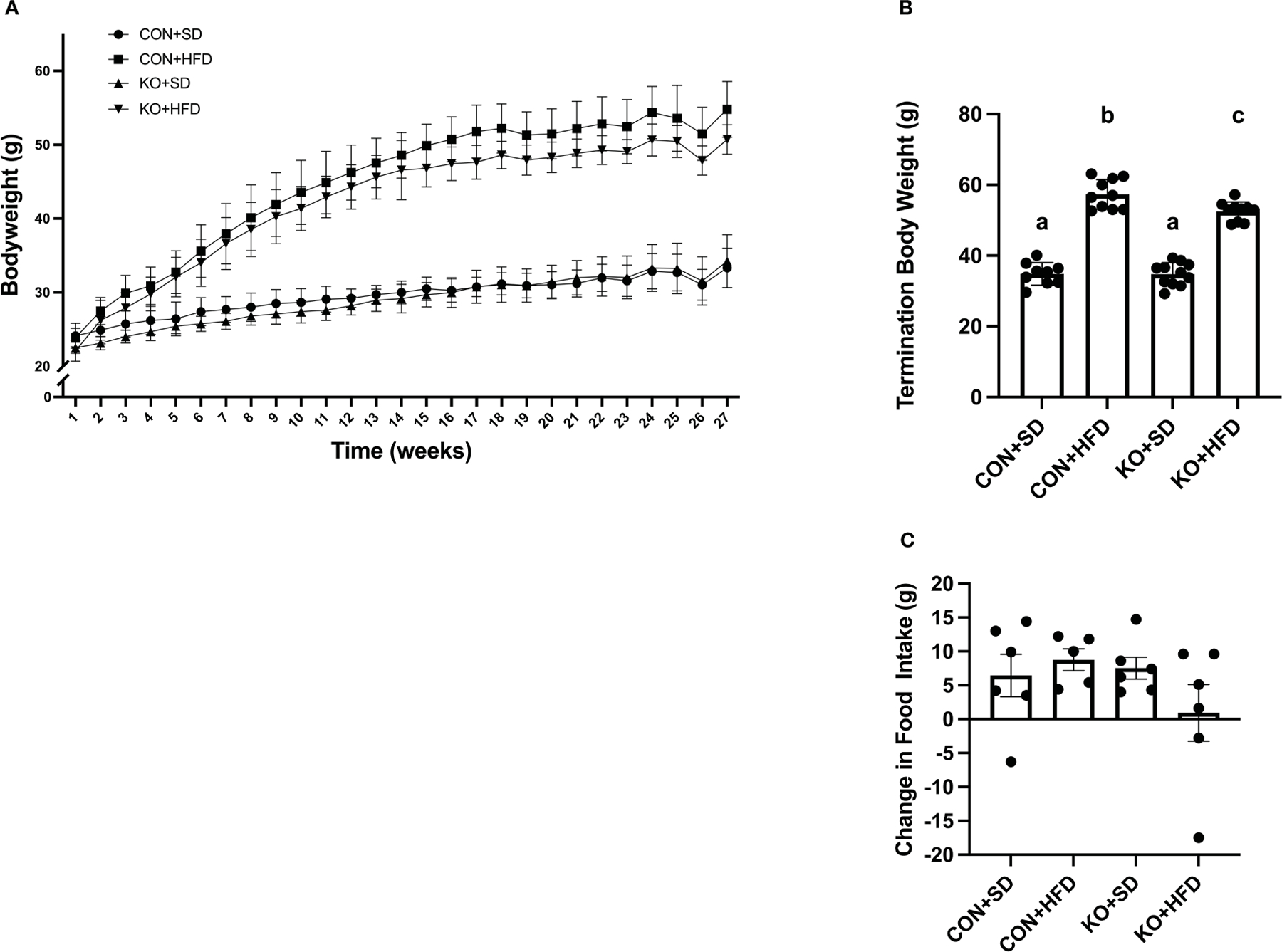
TLR4 ablation leads to less high fat diet-induced weight gain in mice. (**A**) Weekly body weight per group. n=3–12 per group per week. Data is presented as mean ± SD. A linear mixed effect analysis was fit with mice as a random effect and week and group as fixed effects. (**B**) Body weight of mice at termination. n=9–11 per group. Data is presented as mean ± SEM and analysis was performed using one-way ANOVA follwed by Tukey’s multiple comparisons test. (**C**) Change in food intake. n=5–6 cages per group. Data is presented as mean ± SEM and analysis was performed using one-way ANOVA follwed by Tukey’s multiple comparisons test. Different letters indiciate significant differences between groups (p<0.05).

By 4 weeks into the intervention, HFD-fed animals weighed significantly more than SD-fed animals (p<0.01 for all pairwise comparisons between HFD and SD; Figure 1A). At termination, animals on the HFD weighed significantly more than mice fed SD, regardless of genotype (p<0.01 for all pairwise comparisons between HFD and SD groups; Figure 1B); however, the KO+HFD animals weighed significantly less than the CON+HFD animals (52.5 ± 0.8 g. vs. 57.3 ± 1.3g.; p=0.01) while weighing significantly more than both SD groups (p<0.001) (Figure 1B). Changes in body weight could not be explained by changes in food intake, as there were no significant differences in food intake between any two groups (Figure 1C). In general, tissue weights and fat depots differed between diet groups, with no differences observed between CON+HFD compared to KO+HFD (Table 1). Of note, the KO+SD animals had significantly reduced cecum weight and colon length compared to the CON+SD animals, as well as slightly higher mesenteric adipose tissue weight (Table 1).

### Mice exhibit microbial disruption following a HFD, regardless of genotype

**FIGURE 2:**
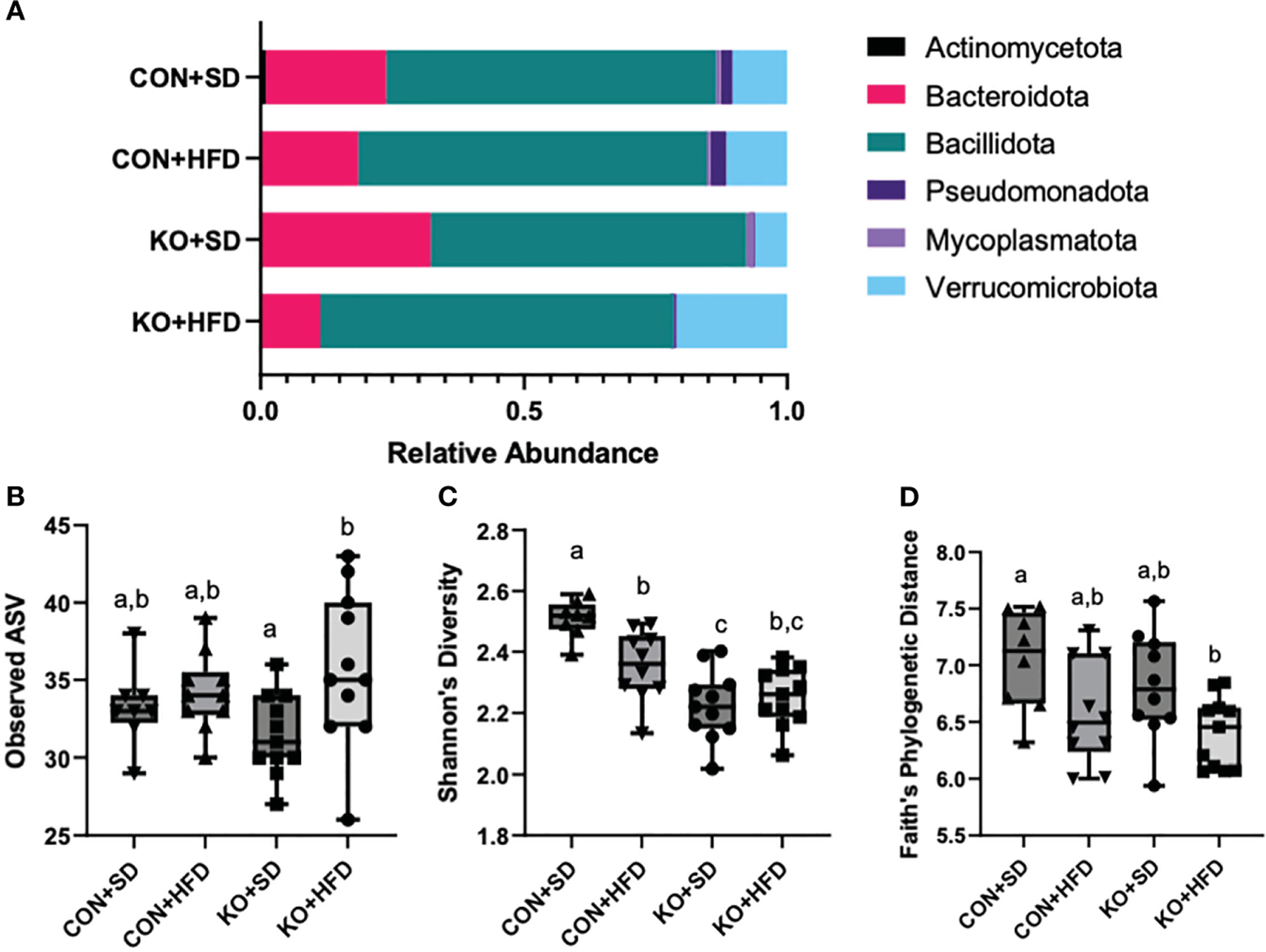
High fat diet induced changes to the microbiota, regardless of genotype. (**A**) Taxa barplots of the average relative abundance of major bacterial phyla across experimental groups. (**B**) Total observed ASVs; (**C**) Shannon’s diversity scores; and (**D**) Faith’s phylogenetic diversity for each experimental group. Box and whisker plots display min, max, median, and 25^th^ and 75^th^ percentile values. Significant difference (p<0.05) are represented by different letters.

We used 16S rRNA sequencing to characterize diet and genotype-associated differences in the microbiota. Using Kruskal-Wallis tests with FDR <0.05, there were significant differences in all six phyla detected (Figure 2A; Table 2). Specifically, Pseudomonadota (formerly Proteobacteria) were associated with genotype and were higher in CON mice compared to KO animals. Bacillidota (formerly Firmicutes) were associated with diet and were higher in HFD-fed animals. Verrucomicrobiota were specifically associated with KO +HFD, and both Actinomycetota (formerly Actinobacteria) and Mycoplasmatota (formerly Tenericutes) were associated with the CON+SD group. Bacteriodota (formerly Bacteroidetes) differences were influenced by both diet and genotype.

In addition to compositional differences between the diet groups, there were also significant differences in several parameters of a-diversity. Specifically, when comparing groups at the genus level, there were significant differences in ASV richness (p-value: 0.041, H=8.264; Figure 2B), Shannon’s diversity (p<0.001, H=21.191; Figure 2C), and Faith’s Phylogenetic indices (p=0.027; H=9.207; Figure 2D), but not in Pileu’s Evenness (p=0.195, H=4.705; data not shown). In general, the most striking differences was in Shannon’s diversity and was driven by higher diversity in the microbiota of the CON+SD group relative to all other groups (Figure 2C).

**FIGURE 3:**
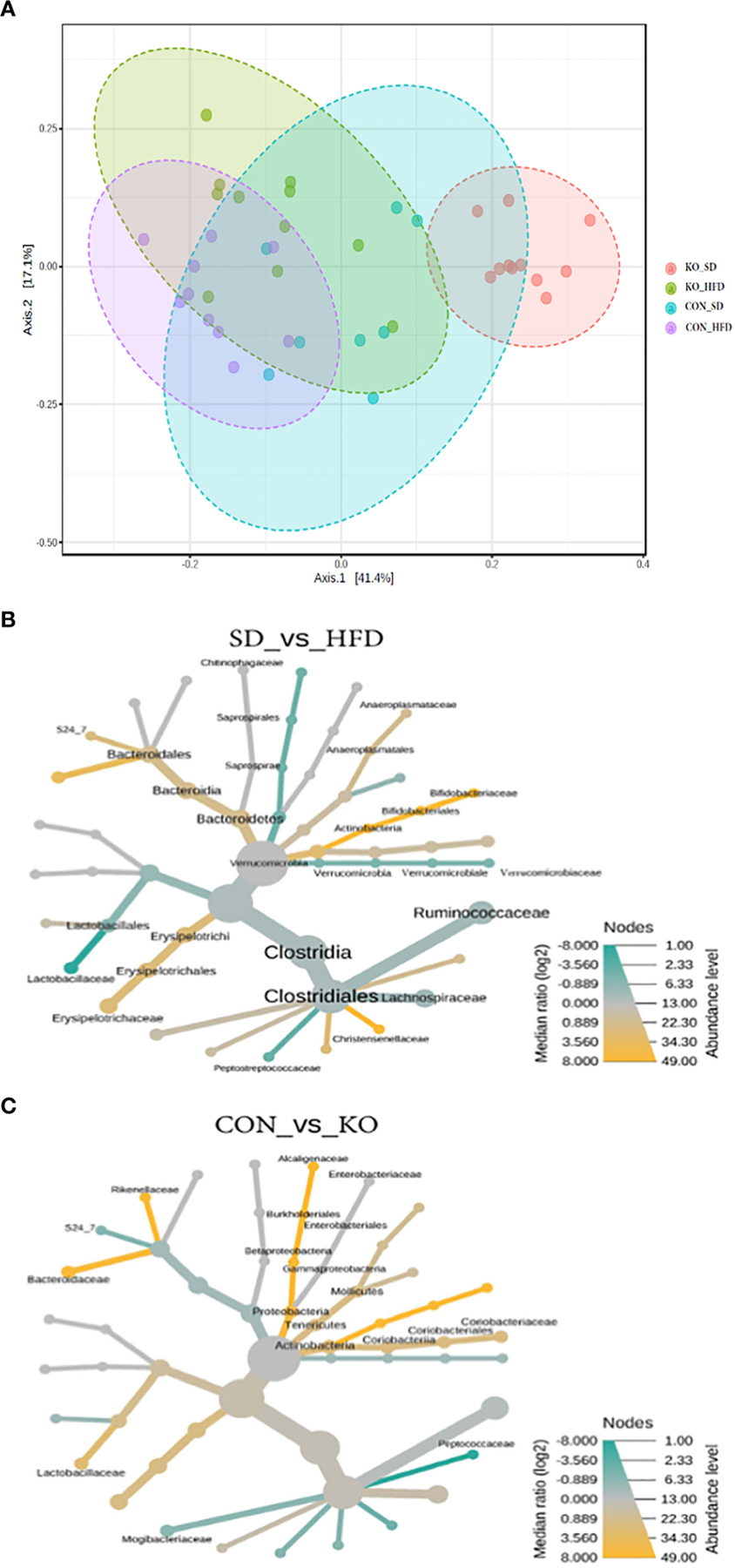
Both diet and genotype shape the gut microbiota. (**A**) A Principle Coordinates Analysis (PCoA) of Bray Curtis distances demonstrates that the TLR4 KO animals have a distinct microbiota from their wild-type littermates when consuming a low fat diet, but that the microbiota of both groups begins to converge with introduction of a HFD. (**B**) Heat tree of differentially abundant taxa by diet group, determined using a non-parametric Wilcoxon test. (**C**) Heat tree of differentially abundant taxa by genotype, determined using a non-parametric Wilcoxon test.

When visualizing the Bray Curtis distances between the four groups on a Principal Coordinates Analysis (PCoA; PERMANOVA F=11.7, R2 = 0.494, p<0.001), the KO+SD forms a cluster that is distinctly separate from the other groups along the first axis, which represents 41.4% of the total diversity (Figure 3A). Introduction of a HFD produces similar shifts in the microbiota of both the CON and KO mice and there is considerable overlap between the two HFD groups, suggesting that although the SD microbiota differed between the CON and KO mice, microbiota disruption was induced in both groups receiving the HFD. There was also a significant difference in the within group variance (PERMDISP, F=3.4905; p= 0.025), suggesting that the variability in microbial profiles between animals was reduced by the HFD.

Heat trees comparing diet groups (Figure 3B) or genotypes (Figure 3C) identified microbes associated with specific conditions. Relative to HFD, the SD-fed mice were enriched in Bifidobacteriacae, Anaeroplasmataceae, Bacteriodales, Christensenellaceae, and Erysipelotrichaceae, but had reduced Lactobacillaceae, Verrucomicrobiaceae, and Peptostreptococcacaceae (Figure 3B). In comparison, the CON mice were enriched in Actinobacteria, Lactobacillaceae, Alcaligenaceae, Bacteriodaceae, and Rickenellaceae relative to KO mice, which were enriched in Peptococcaceae and Mogibacteraceae (Figure 3C).

**FIGURE 4:**
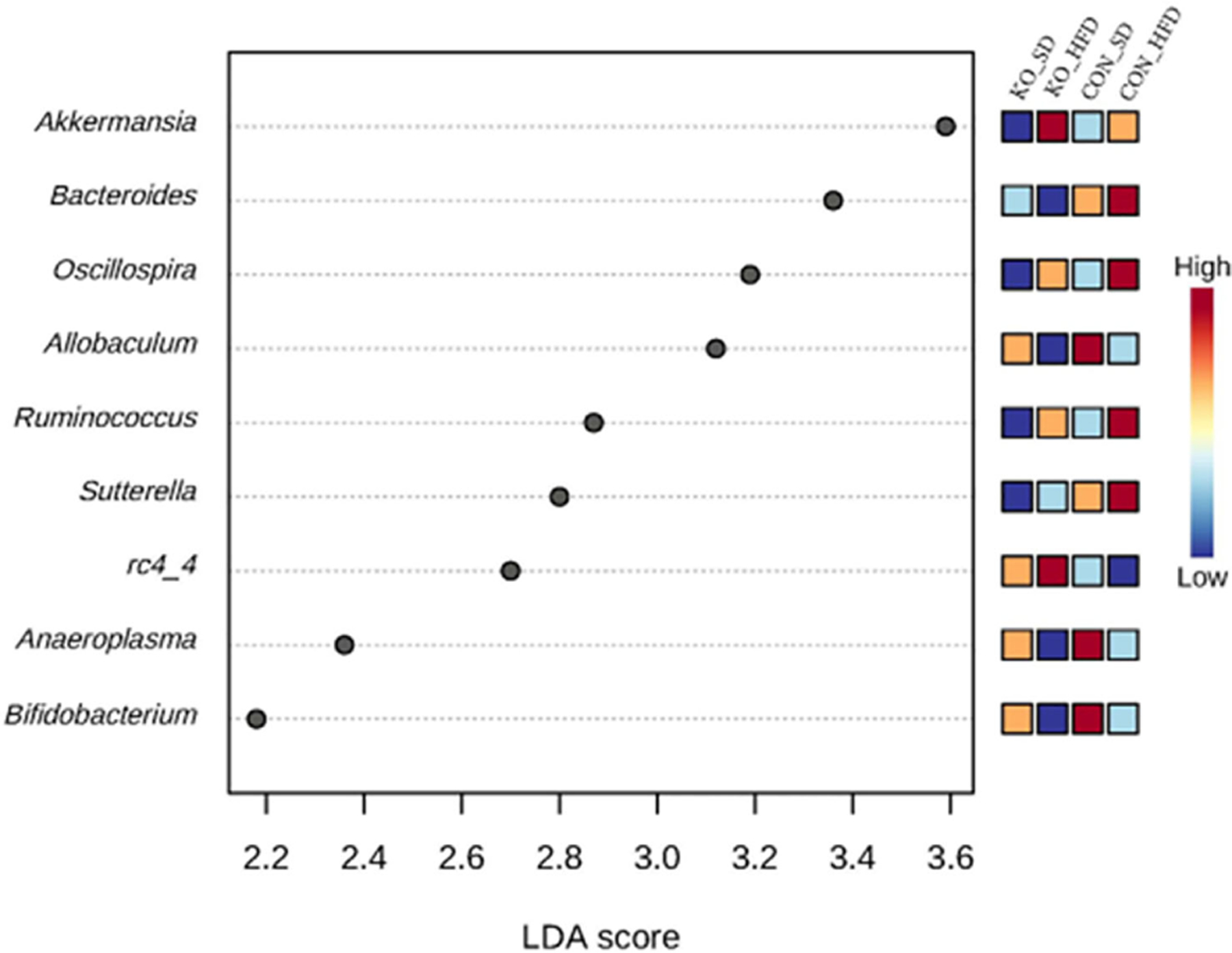
Differentially abundant genera across diet x genotype groups determined using LEfSe. Using the MicrobiomeAnalyst implementation of LeFSe, differentially abundant genera were identified by an FDR adjusted p-value of 0.05 and an LDA score of 2.

Using LefSE, nine genera were identified as biomarkers that distinguish between the treatment groups (Figure 4). Four of these genera: *Ocillospora, Allobaculum, Ruminococcus*, and *Bifidobacterium* were mostly influenced by diet group, while *Bacteroides, Sutterella*, and RC4-4 were linked to genotype (Figure 4). *Akkermansia* and *Anaeroplasma* were specifically enriched in KO+HFD relative to other treatment groups (Supplementary Table S1).

### TLR4 KO mice are susceptible to HFD-induced intestinal impairments, but partially protected from arterial stiffness

**FIGURE 5:**
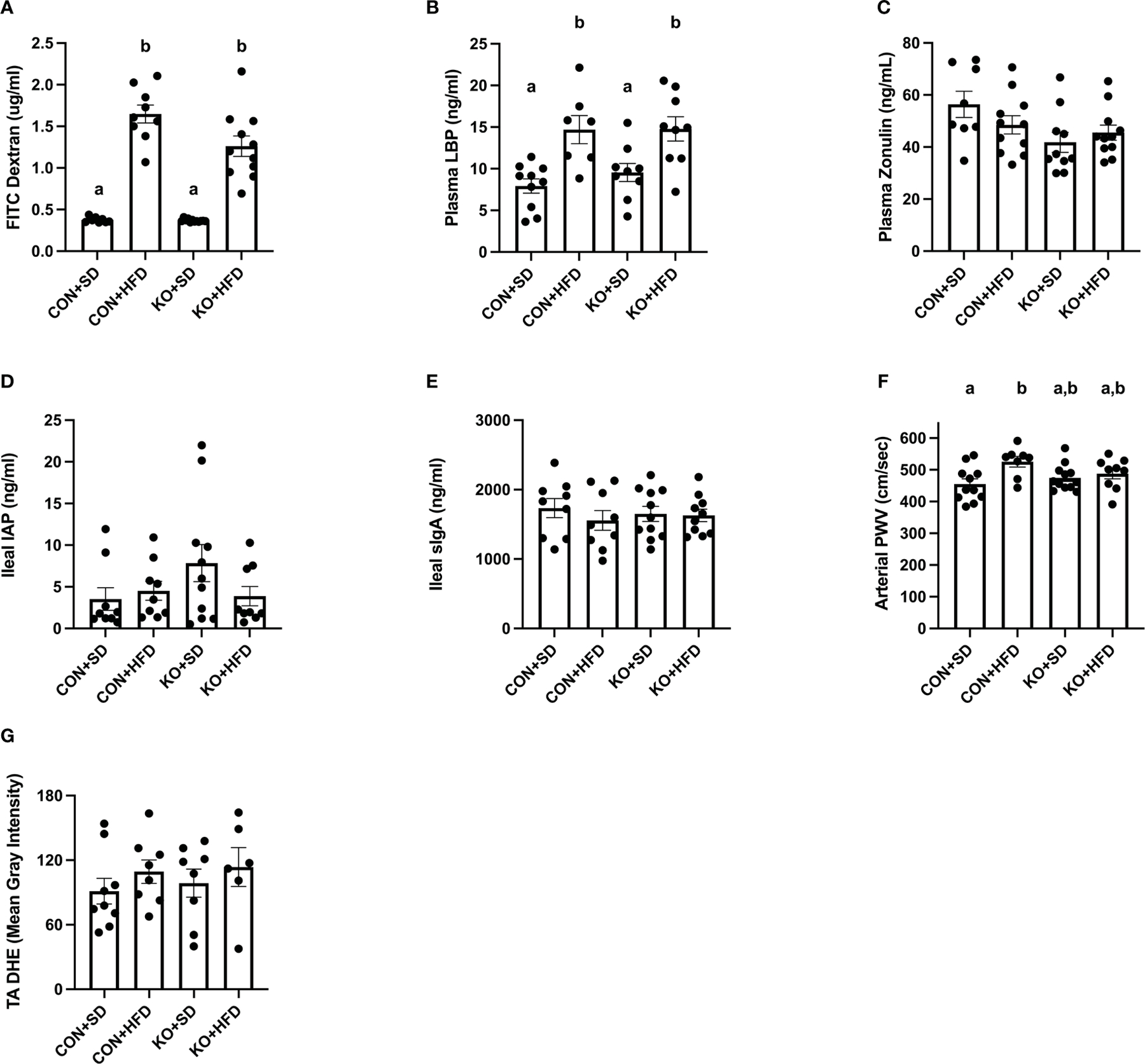
TLR4 ablation partially protects against arterial stiffness. (**A**) Mean plasma FITC Dextran concentration per group. n=9–12 per group. Data is presented as mean ± SEM. Analysis was performed using Welch ANOVA followed by Dunnett’s T3 multiple comparisons test. (**B**) Mean plasma LBP concentration per group. n=7–10 per group. Data is presented as mean ± SEM. Analysis was performed using one-way ANOVA follwed by Tukey’s multiple comparisons test. (**C**) Mean Zonulin concentration per group. n=8–11 per group. Data is presented as mean ± SEM. Analysis was performed using one-way ANOVA follwed by Tukey’s multiple comparisons test. (**D**) Mean ileal IAP concentration per group. n=9–11 per group. Data is presented as mean ± SEM. Analysis was performed using one-way ANOVA follwed by Tukey’s multiple comparisons test. (**E**) Mean sIgA concentration per group. n=9–11 per group. Data is presented as mean ± SEM. Analysis was performed using one-way ANOVA follwed by Tukey’s multiple comparisons test. (**F**) Mean arterial PWV per group. n=8–12 per group. Data is presented as mean ± SEM. Analysis was performed using one-way ANOVA follwed by Tukey’s multiple comparisons test. (**G**) Mean thoracic aorta dihydroethidium intensity per group. n=6–9 per group. Data is presented as mean ± SEM. Analysis was performed using one-way ANOVA. Different letters indiciate significant differences between groups (p<0.05). FITC, fluorescein isothiocyanate; LBP, lipoprotein binding protein; IAP, ileal intestinal alkaline phosphatase; sIgA, secretory immunoglobulin A; PWV, pulse wave velocity; TA, thoracic aorta; DHE, dihydroethidium.

Both CON+HFD and KO+HFD displayed increased intestinal permeability compared to SD-fed animals, indicated by higher circulating levels of FITC after oral gavage (CON+HFD: 1.6 ± 0.1 ug/mL; KO+HFD: 1.3 ± 0.1 ug/mL; CON+SD: 0.4 ± 0.01 ug/mL; KO+SD: 0.4 ± 0.01 ug/mL; p<0.001 for all pairwise comparisons between HFD and SD groups; Figure 5A). Circulating lipopolysaccharide binding protein (LBP), a proxy measure for circulating endotoxin (Schumann and Zweigner, 1999), was also significantly higher in HFD-fed animals regardless of genotype (CON+HFD: 14.7 ± 1.7 ng/mL; KO+HFD: 14.8 ± 1.5 ng/mL; CON+SD: 7.9 ± 0.9 ng/ml; KO+SD: 9.6 ± 1.1 ng/ml; p<0.05 for all pairwise comparisons between HFD and SD groups; Figure 5B). Plasma zonulin (ZO) (more accurately, zonulin-family peptides (Fasano, 2021)), and intestinal alkaline phosphatase (IAP), an enzyme that dephosphorylates LPS (Bates et al., 2007), did not significantly differ between any of the groups (ZO: p=0.08, Figure 5C; IAP: p=0.2, Figure 5D). In addition, secretory immunoglobin (sIgA) measured in ileum was also the same across diet and genotype (p=0.8; Figure 5E).

**FIGURE 6:**
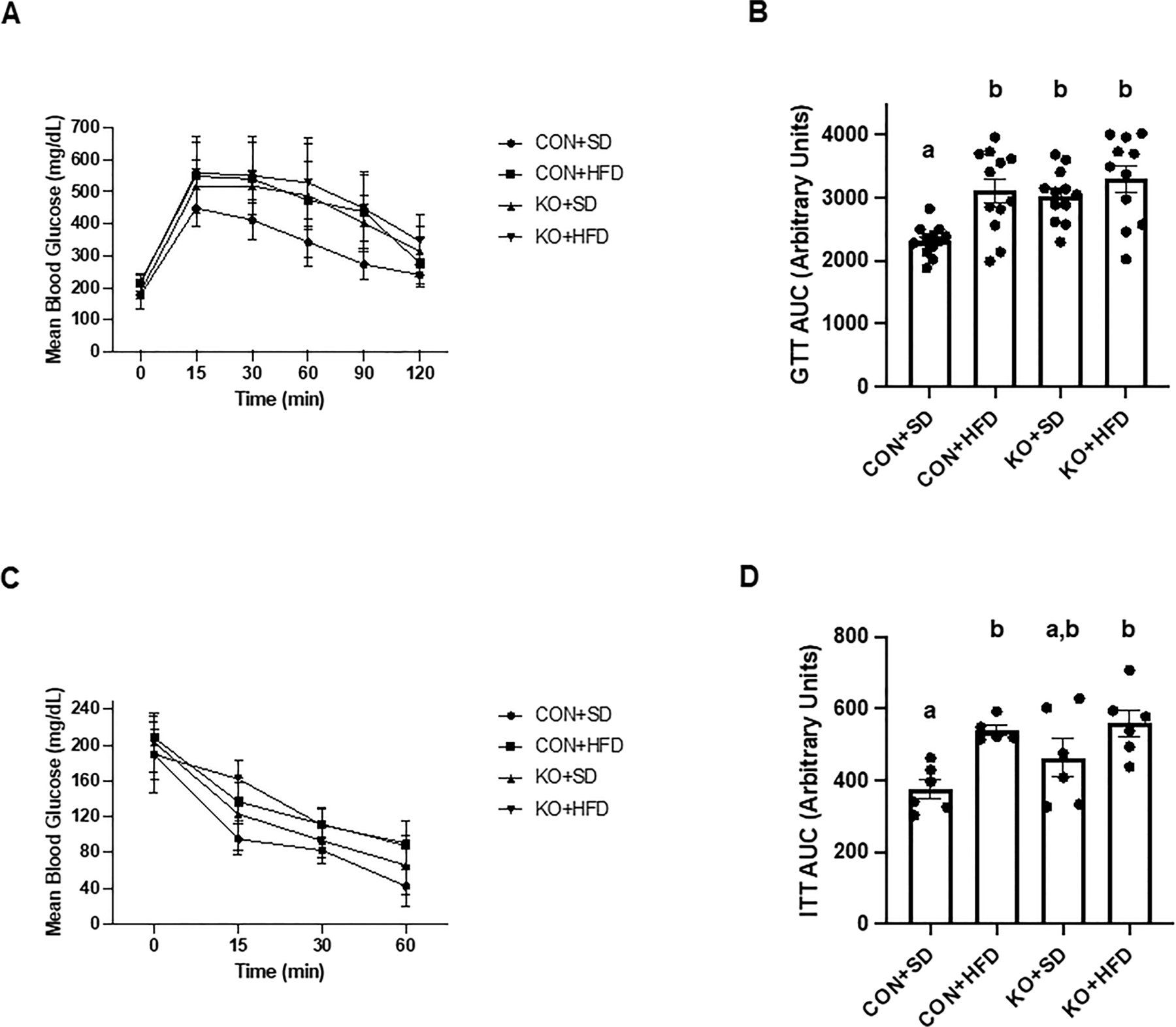
Effect of TLR4 ablation on glucose paramters in SD- and HFD-fed mice. (**A, B**) Glucose tolerance test curves and glucose tolerance test area under the curve (AUC) per group. n=11–12 per group. Curves are presented as mean ± SD. Analysis for curves were performed using a two-way repeated measures ANOVA followed by Tukey’s multiple comparisons test. AUC barplots are presented as mean ± SEM and analysis was performed using Welch ANOVA followed by Dunnett’s T3 multiple comparisons test. (**C, D**) Insulin tolerance test curves and insulin tolerance test AUC per group. n=5–6 per group. Curves are presented as mean ± SD. Analysis for curves were performed using a two-way repeated measures ANOVA followed by Tukey’s multiple comparisons test. AUC barplots are presented as mean ± SEM and analysis was performed using one-way ANOVA follwed by Tukey’s multiple comparisons test. Different letters indiciate significant differences between groups (p<0.05). AUC=area under the curve.

Arterial stiffness was assessed via arterial pulse wave velocity (aPWV). After the 6-month dietary intervention, CON+HFD animals displayed significantly higher aPWV than CON+SD animals (CON+HFD: 525.4 ± 16.5 cm/sec; CON+SD: 455.2 ± 16.5 cm/sec; p=0.02, Figure 5F). However, TLR4 deletion exerted a protective effect against the HFD such that aPWV was not elevated in KO+HFD mice compared to CON+SD or KO+SD, Figure 5F). Thoracic aorta DHE intensity was similar in all groups (p=0.6) (Figure 5G). Glucose response (Figure 6A) and area under the (AUC) (Figure 6B) were significantly higher in all groups compared to the CON+SD cohort. Similarly, insulin responses (Figures 6C, D) were increased in HFD-fed animals compared to CON+SD, while KO+SD was not significantly different from any of the groups.

## Discussion

TLRs are critical for pathogen recognition and immune health. One of the primary ligands for TLR4 is bacterial lipopolysaccharide (LPS), a component of the cell wall of all Gram-negative organisms. Translocation of non-pyrogenic levels of LPS from the intestinal lumen into the circulation occurs when disruptions to the gut microbiota result in reduced integrity of the intestinal barrier, a condition termed “metabolic endotoxemia” (Cani et al., 2007a; Cani et al., 2008). In turn, activation of TLR4 in immune cells and host tissues generates downstream signaling cascades leading to chronic inflammation. This chronic inflammation has been linked to cardiometabolic disruptions, including arterial stiffness (Roman et al., 2005; Mozos et al., 2017). We have demonstrated that suppression of the microbiota reverses arterial stiffness in HFD-fed mice (Battson et al., 2018). Therefore, we hypothesized that animals lacking TLR4 signaling capacity would be protected from the cardiometabolic consequences of a high fat diet, despite experiencing similar perturbations to the gut microbiota.

Interestingly, we found that TLR4 KO mice had a distinct microbial profile compared to CON littermates, but that introduction of a HFD induced similar microbial changes in both genotypes. Differences in the microbiota of TLR4 KO animals compared to CON littermates on a SD included reduced Shannon’s diversity and distinct clustering along the first principal coordinate of an ordination of Bray-Curtis distances. Cuesta et al., recently reported similar patterns of alcohol-induced inflammation in TLR4 KO animals (Cuesta et al., 2021). The authors observed that the microbiota of the two genotypes displayed unique responses to ethanol exposure, while we observed very similar changes in response to HFD feeding.

We found that similarity between the two HFD groups was greater than similarity between the TLR4 KO groups on different diets. Specifically, *Ocillospira*, *Ruminoccocus*, and *Bacteroides* were higher in animals consuming HFD, while *Bifidobacterium* and *Allobaculum* were lower. None of the taxa that were increased with HFD are consistently associated with obesity or inflammation; however, L-methionine production by *Ruminococcus* has been associated with cardiovascular phenotypes in obese humans (Kurilshikov et al., 2019). In contrast, numerous studies have linked reduced *Bifidobacterium* to HFD (Cani et al., 2007b; Wang et al., 2020; Schellekens et al., 2021) and we have previously reported inverse associations between *Bifidobacterium* and vascular function (Battson et al., 2018; Battson et al., 2019; Trikha et al., 2021).

In addition to microbial perturbations, we also observed increased intestinal permeability in animals fed HFD, regardless of genotype. Although TLR4 is expressed in intestinal epithelial cells (IEL), it has been speculated that the constant exposure to intestinal bacterial LPS necessitates tight regulation of TLR4 activation in the lumen (Villena and Kitazawa, 2014). This was confirmed in a recent study by Crame et al., who demonstrated that an intestinal specific KO of TLR4 had no impact on intestinal permeability in the ileum or colon (Crame et al., 2021). Therefore, it is unsurprising that the TLR4 KO offered no protection against HFD-induced intestinal barrier dysfunction.

Numerous studies have reported protective effects of TLR4 deletion following HFD or nutrient excess (Shi et al., 2006; Poggi et al., 2007; Lin et al., 2020). Furthermore, Haimovich et al., demonstrated that poor insulin action and metabolic health are key activators of TLR4 signaling (Zhang et al., 2015; Zhang et al., 2016; Zhang et al., 2019). Thus, it is plausible that deleting TLR4 in mice could help alleviate the resulting inflammatory effects of a HFD on cardiometabolic health. Interestingly, in the current study, TLR4^−/−^ mice did not display improved glucose metabolism following a HFD. Although the reasons for this discrepancy are unclear, it should be noted that some previous studies have also failed to demonstrate a protective effect of TLR4 inhibition on aspects of metabolic function (Dalby et al., 2018; Moser et al., 2018). Fewer studies have examined the effects of TLR4 signaling on vascular function, and to our knowledge, no studies have determined the effects on arterial stiffness. TLR4^−/−^ deletion or inhibition has been shown to improve endothelial dysfunction in mice caused by angiotensin II delivery or type II diabetes (Liang et al., 2013; Hernanz et al., 2015). Interestingly, and in line with the current study, previous reports have suggested that TLR4^−/−^ mice are partially protected from vascular inflammation and atherosclerosis following a HFD (Kim et al., 2007; Ding et al., 2012). Collectively with the current results, these data suggest that diet-induced vascular disturbances are partially, albeit not entirely, mediated by TLR4 signaling.

Limitations of the current study should be noted. First, the TLR4 knockout model was a whole body knock out and thus it is unclear whether the observed protective effect on arterial stiffness was due to local TLR4 signaling in the vasculature or more remote signaling elsewhere. Tang et al., demonstrated that endothelial-specific TLR4 deletion protected mice from cerebral cavernous malformations, a cause of stroke, thus suggesting that endothelial TLR4 signaling may directly drive vascular dysfunction (Tang et al., 2017). Second, only male mice were studied. Given that some effects of TLR4 deficiency have been shown to be sex-dependent (Li et al., 2022; Tirelle et al., 2022), we cannot extrapolate our findings to beyond male mice. In conclusion, we found that TLR4−/− mice are not protected from the intestinal disturbances of a HFD but are partially protected from the downstream consequences within the vasculature.

Ultimately, our results suggest the importance of TLR4 signaling on the gut microbiota and arterial stiffness. Through the observed differences in gut microbial composition between TLR4 KO and CON mice, we highlight TLR4 signaling as a potential mediator of the gut microbial composition while supporting a possible bi-directional relationship between TLR4 signaling and the gut microbiota. Secondly, our data suggest that TLR4 signaling is at least partially responsible for the microbial-provoked arterial stiffening following caloric excess and may play a role in elevated cardiovascular risk associated with obesity, with our future work aiming to understand the specific mechanisms at play.

## Supplementary Material

supplementary table 1

## Figures and Tables

**TABLE 1:** General characteristics of mice at termination.

	CON+SD	CON+HFD	KO+SD	KO+HFD	p-value
Liver weight (mg)*	1368.0 ± 42.8 ^a^	3648.0 ± 237.4^b^	1449.0 ± 86.6^a^	3320.0 ± 191.7^b^	<0.01
Heart weight (mg)*	159.9 ± 6.3^a,b^	189.3 ± 10.1^b^	141.2 ± 3.3^a^	176.7 ± 2.6^b^	<0.01
Spleen weight (mg)^+^	82.6 (20.6) ^a^	127.3 (57.6) ^b^	74.5 (11.5) ^a^	108.6 (6.4)^b^	<0.01
Epi Adipose weight (mg)	1096.0 ± 149.3^a^	1527.0 ± 140.3^a,b^	1582.0 ± 107.6^b^	1217 ± 111.5^a,b^	0.03
SQ Adipose weight (mg)*	350.4 ± 63.0^a^	2094.0 ± 143.3^b^	535.9 ± 45.6^a^	1782.0 ± 70.8^b^	<0.01
PVAT weight (mg)	43.5 ± 8.0^a^	96.5 ± 12.1^b^	53.1 ± 6.1^a^	90.1 ± 8.5^b^	<0.01
(log) MAT weight (mg)	2.6 ± 0.05^a^	3.1 ± 0.04^b^	2.8 ± 0.03^c^	3.0 ± 0.04^b,c^	<0.01
Cecum weight (mg)	395.8 ± 43.0^a^	247.1 ± 20.3^b^	288.8 ± 20.5^b^	241.8 ± 18.3^b^	<0.01
Colon length (cm)	6.0 ± 0.1^a^	5.9 ± 0.1^a,b^	5.4 ± 0.2^b^	5.6 ± 0.1^a,b^	<0.01

n=9–12 per group. Data is presented as mean ± SEM unless otherwise stated. Analysis was performed using one-way ANOVA follwed by Tukey’s multiple comparisons test unless otherwise stated.

* Analysis was performed using Welch ANOVA followed by Dunnett’s T3 multiple comparisons test.

+ Data is presented as median (interquartile range) and analysis was performed using Kruskal Wallis test followed by Dunns multiple comparisons test.

Different letters indiciate significant differences between groups (p<0.05). Epi Adipose, epidydimal adipose tissue; SQ Adipose, subcuteneous adipose tissue; PVAT, perivascular adipose tissue; MAT, mesenteric adipose tissue.

**TABLE 2:** Kruskal-Wallis (*H*) was used to examine differences at the phyla level across experimental groups. A False Discovery Rate (FDR) of <0.05 was considered statistically significant.

Phylum	P Values	FDR	*H* statistic
**Actinomycetota** (formerly Actinobacteria)	0.015	0.018	10.461
**Bacillota** (formerly Firmicutes)	0.002	0.003	15.186
**Bacteroidota** (formerly Bacteroidetes)	0.003	0.005	13.853
**Mycolplasmatota** (formerly Tenericutes)	0.024	0.024	9.439
**Pseudomonadota** (formerly Proteobacteria)	0.001	0.001	19.296
**Verrucomicrobiota**	0.001	0.003	15.698

## Data Availability

The data presented in the study are deposited in the EMBL-ENI repository, accession number PRJEB61188.

## References

[R1] Anderson MJ (2001). A new method for non-parametric multivariate analysis of variance: NON-PARAMETRIC MANOVA FOR ECOLOGY. Austral Ecol. 26, 32–46. doi: 10.1111/j.1442-9993.2001.01070.pp.x

[R2] Bates JM, Akerlund J, Mittge E, and Guillemin K (2007). Intestinal alkaline phosphatase detoxifies lipopolysaccharide and prevents inflammation in zebrafish in response to the gut microbiota. Cell Host Microbe 2, 371–382. doi: 10.1016/j.chom.2007.10.010 18078689 PMC2730374

[R3] Battson ML, Lee DM, Jarrell DK, Hou S, Ecton KE, Weir TL, (2018). Suppression of gut dysbiosis reverses Western diet-induced vascular dysfunction. Am. J. Physiology-Endocrinology Metab. 314, E468–E477. doi: 10.1152/ajpendo.00187.2017 PMC6048388 29351482

[R4] Battson ML, Lee DM, Li Puma LC, Ecton KE, Thomas KN, Febvre HP, (2019). Gut microbiota regulates cardiac ischemic tolerance and aortic stiffness in obesity. Am. J. Physiology-Heart Circulatory Physiol. 317, H1210–H1220. doi: 10.1152/ajpheart.00346.2019 PMC6960779 31559829

[R5] Brown JM, and Hazen SL (2015). The gut microbial endocrine organ: bacterially derived signals driving cardiometabolic diseases. Annu. Rev. Med. 66, 343–359. doi: 10.1146/annurev-med-060513-093205 25587655 PMC4456003

[R6] Cani PD, Amar J, Iglesias MA, Poggi M, Knauf C, Bastelica D, (2007a). Metabolic endotoxemia initiates obesity and insulin resistance. Diabetes 56, 1761–1772. doi: 10.2337/db06-1491 17456850

[R7] Cani PD, Bibiloni R, Knauf C, Waget A, Neyrinck AM, Delzenne NM, (2008). Changes in gut microbiota control metabolic endotoxemia-induced inflammation in high-fat diet–induced obesity and diabetes in mice. Diabetes 57, 1470–1481. doi: 10.2337/db07-1403 18305141

[R8] Cani PD, Neyrinck AM, Fava F, Knauf C, Burcelin RG, Tuohy KM, (2007b). Selective increases of bifidobacteria in gut microflora improve high-Fat-Diet-Induced diabetes in mice through a mechanism associated with endotoxaemia. Diabetologia 50, 2374–2383. doi: 10.1007/s00125-007-0791-0 17823788

[R9] Ciesielska A, Matyjek M, and Kwiatkowska K (2021). TLR4 and CD14 trafficking and its influence on LPS-induced pro-inflammatory signaling. Cell. Mol. Life Sci. 78, 1233–1261. doi: 10.1007/s00018-020-03656-y 33057840 PMC7904555

[R10] Clemente-Postigo M, Queipo-Ortuño MI, Murri M, Boto-Ordoñez M, Perez-Martinez P, Andres-Lacueva C, (2012). Endotoxin increase after fat overload is related to postprandial hypertriglyceridemia in morbidly obese patients. J. Lipid Res. 53, 973–978. doi: 10.1194/jlr.P020909 22394503 PMC3329396

[R11] Crame EE, Bowen JM, Secombe KR, Coller JK, François M, Leifert W, (2021). Epithelial-specific TLR4 knockout challenges current evidence of TLR4 homeostatic control of gut permeability. Inflammation Intest Dis. 6, 199–209. doi: 10.1159/000519200 PMC8739639 35083285

[R12] Cuesta CM, Pascual M, Pérez-Moraga R, Rodríguez-Navarro I, García-García F, Ureña-Peralta JR, (2021). TLR4 deficiency affects the microbiome and reduces intestinal dysfunctions and inflammation in chronic alcohol-fed mice. IJMS 22, 12830. doi: 10.3390/ijms222312830 34884634 PMC8657603

[R13] Dalby MJ, Aviello G, Ross AW, Walker AW, Barrett P, and Morgan PJ (2018). Diet induced obesity is independent of metabolic endotoxemia and TLR4 signalling, but markedly increases hypothalamic expression of the acute phase protein, SerpinA3N. Sci. Rep. 8, 15648. doi: 10.1038/s41598-018-33928-4 30353127 PMC6199263

[R14] David LA, Maurice CF, Carmody RN, Gootenberg DB, Button JE, Wolfe BE, (2014). Diet rapidly and reproducibly alters the human gut microbiome. Nature 505, 559–563. doi: 10.1038/nature12820 24336217 PMC3957428

[R15] Ding Y, Subramanian S, Montes VN, Goodspeed L, Wang S, Han C, (2012). Toll-like receptor 4 deficiency decreases atherosclerosis but does not protect against inflammation in obese low-density lipoprotein receptor–deficient mice. Arterioscler Thromb Vasc Biol 32, 1596–1604. doi: 10.1161/ATVBAHA.112.249847.ATVB 22580897 PMC3748807

[R16] Eckel RH, and Krauss RM (1998). American Heart association call to action: obesity as a major risk factor for coronary heart disease. Circulation 97, 2099–2100. doi: 10.1161/01.CIR.97.21.2099 9626167

[R17] Fasano A (2021). Zonulin measurement conundrum: add confusion to confusion does not lead to clarity. Gut 70, 2007–2008. doi: 10.1136/gutjnl-2020-323367 33177164

[R18] Fuke N, Nagata N, Suganuma H, and Ota T (2019). Regulation of gut microbiota and metabolic endotoxemia with dietary factors. Nutrients 11, 2277. doi: 10.3390/nu11102277 31547555 PMC6835897

[R19] Hernanz R, Martínez-Revelles S, Palacios R, Martín A, Cachofeiro V, Aguado A, (2015). Toll-like receptor 4 contributes to vascular remodelling and endothelial dysfunction in angiotensin II-induced hypertension: TLR4 and vascular damage in hypertension. Br. J. Pharmacol. 172, 3159–3176. doi: 10.1111/bph.13117 25712370 PMC4459031

[R20] Kim F, Pham M, Luttrell I, Bannerman DD, Tupper J, Thaler J, (2007). Toll-like receptor-4 mediates vascular inflammation and insulin resistance in diet-induced obesity. Circ. Res. 100, 1589–1596. doi: 10.1161/CIRCRESAHA.106.142851 17478729

[R21] Kurilshikov A, van den Munckhof ICL, Chen L, Bonder MJ, Schraa K, Rutten JHW, (2019). Gut microbial associations to plasma metabolites linked to cardiovascular phenotypes and risk: a cross-sectional study. Circ. Res. 124, 1808–1820. doi: 10.1161/CIRCRESAHA.118.314642 30971183

[R22] Laugerette F, Vors C, Géloën A, Chauvin M-A, Soulage C, Lambert-Porcheron S, (2011). Emulsified lipids increase endotoxemia: possible role in early postprandial low-grade inflammation. J. Nutr. Biochem. 22, 53–59. doi: 10.1016/j.jnutbio.2009.11.011 20303729

[R23] Lee DM, Battson ML, Jarrell DK, Hou S, Ecton KE, Weir TL, (2018). SGLT2 inhibition via dapagliflozin improves generalized vascular dysfunction and alters the gut microbiota in type 2 diabetic mice. Cardiovasc. Diabetol. 17, 62. doi: 10.1186/s12933-018-0708-x 29703207 PMC5921754

[R24] Lee DM, Ecton KE, Trikha SRJ, Wrigley SD, Thomas KN, Battson ML, (2020). Microbial metabolite indole-3-Propionic acid supplementation does not protect mice from the cardiometabolic consequences of a Western diet. Am. J. Physiology-Gastrointestinal Liver Physiol. 319, G51–G62. doi: 10.1152/ajpgi.00375.2019 PMC7468755 32421360

[R25] Li Y, Zhu S, Xie K, Feng X, Chen L, Wu X, (2022). TLR4 in Tph2 neurons modulates anxiety-related behaviors in a sex-dependent manner. Neuropharmacology 216, 109175. doi: 10.1016/j.neuropharm.2022.109175 35787402

[R26] Liang C-F, Liu JT, Wang Y, Xu A, and Vanhoutte PM (2013). Toll-like receptor 4 mutation protects obese mice against endothelial dysfunction by decreasing NADPH oxidase isoforms 1 and 4. Arterioscler Thromb Vasc Biol 33, 777–784. doi: 10.1161/ATVBAHA.112.301087.ATVB 23413427

[R27] Lin H-Y, Weng S-W, Shen F-C, Chang Y-H, Lian W-S, Hsieh C-H, (2020). Abrogation of toll-like receptor 4 mitigates obesity-induced oxidative stress, proinflammation, and insulin resistance through metabolic reprogramming of mitochondria in adipose tissue. Antioxidants Redox Signaling 33, 66–86. doi: 10.1089/ars.2019.7737 31950846

[R28] Moser VA, Uchoa MF, and Pike CJ (2018). TLR4 inhibitor TAK-242 attenuates the adverse neural effects of diet-induced obesity. J. Neuroinflamm. 15, 306. doi: 10.1186/s12974-018-1340-0 PMC6217784 30396359

[R29] Mozos I, Malainer C, Horbańczuk J, Gug C, Stoian D, Luca CT, (2017). Inflammatory markers for arterial stiffness in cardiovascular diseases. Front. Immunol. 8. doi: 10.3389/fimmu.2017.01058 PMC5583158 28912780

[R30] Ojeda P, Bobe A, Dolan K, Leone V, and Martinez K (2016). Nutritional modulation of gut microbiota — the impact on metabolic disease pathophysiology. J. Nutr. Biochem. 28, 191–200. doi: 10.1016/j.jnutbio.2015.08.013 26372091 PMC4757500

[R31] Poggi M, Bastelica D, Gual P, Iglesias MA, Gremeaux T, Knauf C, (2007). C3H/HeJ mice carrying a toll-like receptor 4 mutation are protected against the development of insulin resistance in white adipose tissue in response to a high-fat diet. Diabetologia 50, 1267–1276. doi: 10.1007/s00125-007-0654-8 17426960

[R32] Qiao Y, Sun J, Ding Y, Le G, and Shi Y (2013). Alterations of the gut microbiota in high-fat diet mice is strongly linked to oxidative stress. Appl. Microbiol. Biotechnol. 97, 1689–1697. doi: 10.1007/s00253-012-4323-6 22948953

[R33] Roman MJ, Devereux RB, Schwartz JE, Lockshin MD, Paget SA, Davis A, (2005). Arterial stiffness in chronic inflammatory diseases. Hypertension 46, 194–199. doi: 10.1161/01.HYP.0000168055.89955.db 15911740

[R34] Ryu J-K, Kim SJ, Rah S-H, Kang JI, Jung HE, Lee D, (2017). Reconstruction of LPS transfer cascade reveals structural determinants within LBP, CD14, and TLR4-MD2 for efficient LPS recognition and transfer. Immunity 46, 38–50. doi: 10.1016/j.immuni.2016.11.007 27986454

[R35] Schellekens H, Torres-Fuentes C, van de Wouw M, Long-Smith CM, Mitchell A, Strain C, (2021). Bifidobacterium longum counters the effects of obesity: partial successful translation from rodent to human. EBioMedicine 63, 103176. doi: 10.1016/j.ebiom.2020.103176 33349590 PMC7838052

[R36] Schumann RR, and Zweigner JA (1999). Novel acute-phase marker: lipopolysaccharide binding protein (LBP). cclm 37, 271–274. doi: 10.1515/CCLM.1999.047 10353471

[R37] Segata N, Izard J, Waldron L, Gevers D, Miropolsky L, Garrett WS, (2011). Metagenomic biomarker discovery and explanation. Genome Biol. 12, R60. doi: 10.1186/gb-2011-12-6-r60 21702898 PMC3218848

[R38] Shi H, Kokoeva MV, Inouye K, Tzameli I, Yin H, and Flier JS (2006). TLR4 links innate immunity and fatty acid–induced insulin resistance. J. Clin. Invest. 116, 3015–3025. doi: 10.1172/JCI28898 17053832 PMC1616196

[R39] Tajik N, Frech M, Schulz O, Schälter F, Lucas S, Azizov V, (2020). Targeting zonulin and intestinal epithelial barrier function to prevent onset of arthritis. Nat. Commun. 11, 1995. doi: 10.1038/s41467-020-15831-7 32332732 PMC7181728

[R40] Tang AT, Choi JP, Kotzin JJ, Yang Y, Hong CC, Hobson N, (2017). Endothelial TLR4 and the microbiome drive cerebral cavernous malformations. Nature 545, 305–310. doi: 10.1038/nature22075 28489816 PMC5757866

[R41] Tirelle P, Salaün C, Kauffmann A, Bôle-Feysot C, Guérin C, Huré M, (2022). Intestinal epithelial toll-like receptor 4 deficiency modifies the response to the activity-based anorexia model in a sex-dependent manner: a preliminary study. Nutrients 14, 3607. doi: 10.3390/nu14173607 36079861 PMC9460860

[R42] Trikha SRJ, Lee DM, Ecton KE, Wrigley SD, Vazquez AR, Litwin NS, (2021). Transplantation of an obesity-associated human gut microbiota to mice induces vascular dysfunction and glucose intolerance. Gut Microbes 13, 1940791. doi: 10.1080/19490976.2021.1940791 34313540 PMC8317959

[R43] Villena J, and Kitazawa H (2014). Modulation of intestinal TLR4-inflammatory signaling pathways by probiotic microorganisms: lessons learned from lactobacillus jensenii TL2937. Front. Immunol. 4. doi: 10.3389/fimmu.2013.00512 PMC3890654 24459463

[R44] Vlachopoulos C, Aznaouridis K, and Stefanadis C (2010). Prediction of cardiovascular events and all-cause mortality with arterial stiffness. J. Am. Coll. Cardiol. 55, 1318–1327. doi: 10.1016/j.jacc.2009.10.061 20338492

[R45] Wang B, Kong Q, Li X, Zhao J, Zhang H, Chen W, (2020). High-fat diet increases gut microbiota biodiversity and energy expenditure due to nutrient difference. Nutrients 12, 3197. doi: 10.3390/nu12103197 33092019 PMC7589760

[R46] Zhang Z, Amorosa LF, Coyle SM, Macor MA, Birnbaum MJ, Lee LY, (2016). Insulin-dependent regulation of MTORC2-Akt-FoxO suppresses TLR4 signaling in human leukocytes: relevance to type 2 diabetes. Diabetes 65, 2224–2234. doi: 10.2337/db16-0027 27207509

[R47] Zhang Z, Amorosa LF, Coyle SM, Macor MA, Lubitz SE, Carson JL, (2015). Proteolytic cleavage of AMPKa and intracellular MMP9 expression are both required for TLR4-mediated MTORC1 activation and HIF-1a expression in leukocytes. J Immunol 195, 2452–2460. doi: 10.4049/jimmunol.1500944. J.I. 26232429 PMC4546925

[R48] Zhang Z, Amorosa LF, Petrova A, Coyle S, Macor M, Nair M, (2019). TLR4 counteracts BVRA signaling in human leukocytes via differential regulation of AMPK, MTORC1 and MTORC2. Sci. Rep. 9, 7020. doi: 10.1038/s41598-019-43347-8 31065010 PMC6504875

