# Supplementary material for: Toll-like receptor 4 deletion partially protects mice from high fat diet-induced arterial stiffness despite perturbation to the gut microbiota
Source: Front Microbiomes. 2023 May 23;2:1095997. doi: 10.3389/frmbi.2023.1095997 (PMC11423633; doi:10.3389/frmbi.2023.1095997)
Supplement: Supplementary file 1 [file Table_1.docx]

**Supplemental Table 1.** Taxa that are significantly driving differences among treatment groups as determined by LDA.

| Genera | Pvalues | FDR | KO_SD | KO_HFD | CON_SD | CON_HFD | LDAscore |
| --- | --- | --- | --- | --- | --- | --- | --- |
| Akkermansia | 1.31E-03 | 0.005 | 1904.5 | 9706.5 | 3824.9 | 4978.1 | 3.59 |
| Allobaculum | 6.31E-03 | 0.015 | 596.64 | 449.36 | 3076.5 | 472.2 | 3.12 |
| Anaeroplasma | 2.28E-02 | 0.046 | 472.45 | 18.91 | 330.75 | 243.6 | 2.36 |
| Bacteroides | 7.76E-06 | 2.019E-04 | 109.18 | 462.27 | 2429.5 | 4659.4 | 3.36 |
| Bifidobacterium | 7.60E-05 | 0.001 | 53.727 | 0.55 | 301.88 | 12.2 | 2.18 |
| Oscillospira | 3.18E-04 | 0.001 | 3614.8 | 6482.1 | 4454.9 | 6733.5 | 3.19 |
| rc4_4 | 1.42E-04 | 0.001 | 973.82 | 1137.1 | 179.25 | 142 | 2.7 |
| Ruminococcus | 4.11E-05 | 0.001 | 1059.5 | 2537.2 | 1362.2 | 2532.1 | 2.87 |
| Sutterella | 1.36E-04 | 0.001 | 26.455 | 165.64 | 790.25 | 1288.1 | 2.8 |
